# Effect of Choline Forms and Gut Microbiota Composition on Trimethylamine-*N*-Oxide Response in Healthy Men

**DOI:** 10.3390/nu12082220

**Published:** 2020-07-25

**Authors:** Clara E. Cho, Niklas D. J. Aardema, Madison L. Bunnell, Deanna P. Larson, Sheryl S. Aguilar, Janet R. Bergeson, Olga V. Malysheva, Marie A. Caudill, Michael Lefevre

**Affiliations:** 1 Department of Nutrition, Dietetics and Food Sciences, Utah State University, Logan, UT 84322, USA; niklas.aardema@usu.edu (N.D.J.A.); maddielbunnell@gmail.com (M.L.B.); dpassarolarson@gmail.com (D.P.L.); sheryl.aguilar@usu.edu (S.S.A.); janet.bergeson@usu.edu (J.R.B.); michael.lefevre@usu.edu (M.L.); 2 Division of Nutritional Sciences, Cornell University, Ithaca, NY 14853, USA; ovm4@cornell.edu (O.V.M.); mac379@cornell.edu (M.A.C.)

**Keywords:** dietary precursor intake, choline, gut microbiota, metabolism, trimethylamine-*N*-oxide

## Abstract

Background: Trimethylamine-*N*-oxide (TMAO), a choline-derived gut microbiota-dependent metabolite, is a newly recognized risk marker for cardiovascular disease. We sought to determine: (1) TMAO response to meals containing free versus lipid-soluble choline and (2) effects of gut microbiome on TMAO response. Methods: In a randomized, controlled, double-blinded, crossover study, healthy men (*n* = 37) were provided meals containing 600 mg choline either as choline bitartrate or phosphatidylcholine, or no choline control. Results: Choline bitartrate yielded three-times greater plasma TMAO AUC (*p* = 0.01) and 2.5-times greater urinary TMAO change from baseline (*p* = 0.01) compared to no choline and phosphatidylcholine. Gut microbiota composition differed (permutational multivariate analysis of variance, PERMANOVA; *p* = 0.01) between high-TMAO producers (with ≥40% increase in urinary TMAO response to choline bitartrate) and low-TMAO producers (with <40% increase in TMAO response). High-TMAO producers had more abundant lineages of *Clostridium* from *Ruminococcaceae* and *Lachnospiraceae* compared to low-TMAO producers (analysis of composition of microbiomes, ANCOM; *p* < 0.05). Conclusion: Given that phosphatidylcholine is the major form of choline in food, the absence of TMAO elevation with phosphatidylcholine counters arguments that phosphatidylcholine should be avoided due to TMAO-producing characteristics. Further, development of individualized dietary recommendations based on the gut microbiome may be effective in reducing disease risk

## 1. Introduction

Choline serves as a dietary precursor for the gut microbial-generated trimethylamine (TMA) that is subsequently oxidized by the hepatic enzyme flavin-containing monooxygenase 3 (FMO3) to form trimethylamine-*N*-oxide (TMAO), a newly recognized risk marker for cardiovascular disease [1,2]. Choline is present as free and lipid-soluble forms (mainly as phosphatidylcholine), with food products consisting of various forms of choline [3]. Free choline is absorbed in the small intestine by sodium-independent carrier mediated transport [4]. In contrast, phosphatidylcholine is mainly hydrolyzed to lysophosphatidylcholine by the action of pancreatic phospholipase A2 IB and jejunoileal brush-border phospholipase B, with a portion reacylated to phosphatidylcholine for incorporation into chylomicrons and the remainder further hydrolyzed to glycerophosphocholine (subsequently to glycerophosphate and free choline) [4]. These differences in absorption suggest that free choline, but not phosphatidylcholine, may be a more readily available substrate for gut microbes to form TMA. Consistent with this, free choline, but not phosphatidylcholine, has been shown to yield high urinary TMA excretion in healthy volunteers [5] and fishy odor (from over-production of TMA) in patients with Huntington’s disease [6]. However, recent studies have reported greater TMAO concentrations with the ingestion of ≥2 eggs (consumption of mostly phosphatidylcholine) [1,7], making unclear the contribution of different forms of choline on TMAO production. Further, considerable interindividual variations were found in circulating and urinary TMAO response to supplemental choline [8] and egg ingestion [7,9], but limited attention has been given to the relationship between underlying gut microbiota differences between individuals and TMAO production.

Therefore, we compared acute TMAO response to meals containing supplemental choline bitartrate versus phosphatidylcholine (choline bitartrate and phosphatidylcholine being two of several applicable choline-delivering compounds) and determined whether differences in TMAO response arise due to the gut microbiota differences between individuals. We have previously reported that individuals with a greater ratio of the gut microbial phyla *Firmicutes* to *Bacteroidetes* and a less diverse gut microbiota exhibited higher TMAO response to egg ingestion [9]. In the following randomized, controlled, double-blinded, crossover study comparing choline bitartrate and phosphatidylcholine, we hypothesized that choline bitartrate would elevate TMAO response more than phosphatidylcholine, and that this response would be modified by gut microbiota composition. Because a greater enrichment of *Firmicutes* versus *Bacteroidetes* has been associated with obesity [10], our original study design included both obese and normal-weight individuals to explore differences in gut microbiota composition as a primary determinant of TMAO levels.

## 2. Materials and Methods

### 2.1. Subjects

**Figure 1:**
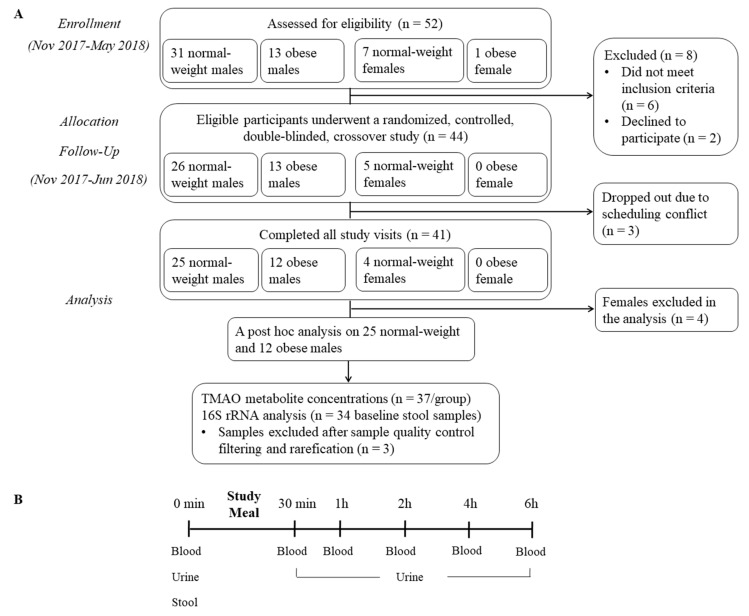
A schematic of the participant flow (**A**) and the study protocol (**B**).

Forty-one (37 men and 4 non-pregnant/non-lactating women) healthy individuals aged 21–50 years completed the study, consisting of 29 individuals (25 men and 4 women) with a BMI of 20–24.9 kg/m^2^ and 12 individuals (12 men and no women) with a BMI of 30–39.9 kg/m^2^. Three individuals (1 normal-weight male, 1 normal-weight female and 1 obese male) were unable to complete the study due to a scheduling conflict. They were recruited through fliers posted on the campus of Utah State University from November 2017 to May 2018. The exclusion criteria were age > 50 y, BMI < 20, 25–29.9 or ≥40 kg/m^2^, pregnant or planning to become pregnant during the course of the study (women), vegetarians, smokers or recreational drug users, individuals with gastrointestinal diseases or complaints, chronic illnesses or other metabolic diseases (including trimethylaminuria), abnormal blood chemistry or blood cell count values indicative of organ dysfunctions, subjects who have taken antibiotics within the past 2 months and those who are unwilling to discontinue pre- and probiotics and dietary supplements for the time leading up to two months before and during the study. Written informed consent was obtained from all participants prior to study enrollment. Figure 1A shows a schematic of the participant flow. 

Clinical Trials Registry: NCT04255368 at https://clinicaltrials.gov/ct2/show/NCT04255368?term=NCT04255368&draw=2&rank=1.

Given difficulty in recruiting obese individuals and women, a revised post hoc calculation was performed based on the achieved sample number and the assumed within-subject coefficient of variations of 20% for plasma TMAO concentrations based on our prior study [9] with duplicate measures at the end of each intervention; α < 0.05 for 2-sided tests and using no corrections for multiple testing. The sample size of 37 participants provided an 80% probability of detecting a difference in TMAO of 10%, and additionally assumed a 10% study drop-out rate. The study was approved by the Institutional Review Board at Utah State University (Protocol ID#: 8547). This trial was registered at clinicaltrials.gov as NCT04255368.

### 2.2. Design

The study design was a randomized, controlled, double-blinded, crossover dietary intervention that provided meals containing 600 mg choline either as choline bitartrate (Balchem, New Hampton, NY, USA; choline bitartrate consisting of 41% choline) or phosphatidylcholine (American Lecithin Company, Oxford, CT, USA; Alcolec 40P consisting of 40% phosphatidylcholine), or no choline control. Both choline bitartrate and phosphatidylcholine were in the form of odorless powder and were adjusted to provide 600 mg choline (choline bitartrate provided ~40% choline by weight; phosphatidylcholine derived from soybean provided ~15% choline by weight). The study meals were prepared by mixing 118 mL of condensed tomato soup with water to a total volume of 237 mL, heating in a commercial microwave for 2.5 min on high and cooled for 1 min to a serving temperature at 55–58 °C, then adding the respective choline-containing powders. 

The order of three study meals was determined by a study investigator using a random number generator (random.org), and the participants were randomized to one of six meal sequences A through F. Approximately the same number of participants were assigned to each of the six meal sequences (out of the 37 participants, *n* = 5 for sequences A and C, *n* = 6 for sequence B and *n* = 7 for sequences D–F). Each meal was administered in a single day separated by a one-week washout period. Participants as well as study personnel who interacted with participants or analyzed the samples were not provided with information linking the study meal order and content (i.e., double-blinded). All meals were prepared in the Center for Human Nutrition Studies Clinic kitchen at Utah State University on the morning of testing.

### 2.3. Protocol

After a 10-h overnight fast, participants arrived at the Center for Human Nutrition Studies Clinic at Utah State University between 0700–1000 for each of the three visits. The day prior to the session, participants were advised to avoid consumption of grapefruit juice and indole-containing vegetables (e.g., broccoli, brussels sprouts, cabbage) [11] and fish [9] as they influence FMO3 activity and/or TMAO response. A 24-h food diary was used to assess compliance to the grapefruit juice, indole food and fish restriction. A baseline blood sample was obtained by a phlebotomist using a standard venipuncture procedure and participants collected their baseline urine. Participants were also asked to submit a one-time baseline stool sample. Participants were provided with a meal consisting of one cup (237 mL) of tomato soup containing randomly allocated choline bitartrate, phosphatidylcholine or no choline control, a bagel with margarine-butter spread and one cup of water. Meals were consumed within a 15-min period. Following the study meal consumption, serial blood was obtained at 30 min, and 1, 2, 4 and 6 h, and each participant collected their urine sample throughout the 6-h study period; this timeframe was based on our previous studies demonstrating that 6 h were sufficient to capture the times where the change in TMAO was the greatest and reached steady state [12]. At 4.5 h, participants were provided with a fixed snack (i.e., apple sauce) and water. Throughout each study session, participants refrained from eating and drinking foods and beverages (other than water) outside those provided by study personnel. The study protocol is shown in Figure 1B.

### 2.4. Sample Collection

#### 2.4.1. Serum and Whole Blood

At the screening visit, whole blood was collected in serum separator and EDTA-coated tubes (Becton, Dickinson and Company, Franklin Lakes, NJ, USA) for blood chemistry profile and complete blood count, respectively. 

#### 2.4.2. Plasma and Buffy Coat

For each study session, whole blood was collected in EDTA-coated tubes, placed on ice immediately and centrifuged at 2000× *g* at 4 °C for 10 min. Plasma was aliquoted into screw-top cryogenic vials and stored at −80 °C for further metabolite analyses. Buffy coat was aliquoted into screw-top cryogenic vials (Thermo Fisher Scientific, Waltham, MA, USA) with 50 µL DMSO, gently mixed by inversion and stored at −80 °C for genotyping of the flavin-containing monooxygenase isoform 3 (*FMO3* G472A rs 2266782; E158K) genetic variant known to influence TMA conversion. 

#### 2.4.3. Urine

At study baseline, spot urine was self-collected in wide-mouth specimen containers (120 mL; Thermo Fisher Scientific). In addition, 6-h urine was self-collected in wide-mouth polyethylene bottles (1 L; Thermo Fisher Scientific) throughout the study period. The total urine volume was recorded for study baseline and the 6-h study period. Urine was kept on ice prior to aliquoting into screw-top cryogenic vials for storage at −80 °C for further metabolite analyses. 

#### 2.4.4. Stool

A ~40 g stool sample from a single bowel movement was self-collected using a stool collection kit containing a specimen receptacle, disposable sterile spoon, sterile conical tubes (BD) and disposable exam gloves. Participants delivered their stool samples in a thermo-insulated bag with ice packs to the Center for Human Nutrition Studies Clinic. Stool samples were aliquoted and stored at −80 °C for further gut microbiome analyses.

#### 2.4.5. Study Meal Homogenate

Each study meal was homogenized in a commercial blender, aliquoted into storage tubes and stored at −80 °C for metabolite analyses. 

### 2.5. Analytical Methodology

#### 2.5.1. Screening Blood Chemistry and Complete Cell Counts

Serum concentrations of fasting glucose, blood urea nitrogen (BUN), creatinine (Cr), total bilirubin, total protein, alkaline phosphatase (ALP), aspartate aminotransferase (AST) and alanine aminotransferase (ALT), as well as complete blood cell counts of red blood cells, white blood cells, lymphocytes, monocytes and neutrophils, were measured from blood collected at screening (LabCorp, Phoenix, AZ, USA). The estimated glomerular filtrate rate (eGFR) was calculated using the Chronic Kidney Disease Epidemiology Collaboration equation [13]. 

#### 2.5.2. TMAO and Choline Measurements in Study Meal, Plasma and Urine

TMAO and choline were measured in study food, plasma and urine samples by liquid chromatography-tandem mass spectrometry (LC-MS/MS) as previously described [9,14] with modifications from [15]. Urinary metabolite concentrations were adjusted for creatinine, which was measured using a Creatinine (urinary) Colorimetric Assay Kit (Cayman Chemical, Ann Arbor, MI, USA) according to the manufacturer’s specifications. Study meal total choline (sum of free choline, glycerophosphocholine, phosphocholine, phosphatidylcholine, sphingomyelin) and betaine contents were quantified [16,17] with modifications [18]. The system consisted of a TSQ Quantum Ultra mass spectrometer with electrospray ionization source equipped with a refrigerated Accela autosampler and an Accela pump with degasser (Thermo Fisher Scientific). Samples were run in batches with each batch containing all time points and study meals, with an equal number of samples from each study meal group.

#### 2.5.3. 16S rRNA Gene Sequencing

Genomic DNA was extracted from ~100 mg of stool using the QIAmp Fast DNA Stool Mini Kit (Qiagen, Valencia, CA, USA) with bead beating according to the manufacturer’s specifications. The DNA concentration was quantified using the NanoQuant Plate spectrophotometer (Tecan, Männedorf, Switzerland) and normalized to a concentration of 5 ng/µL, and the DNA quality was assessed on a 1.5% agarose gel. The V4 hypervariable region of the bacterial 16S rRNA gene was polymerase chain reaction (PCR) amplified and sequenced with the 16S-specific primers fused to the standard Illumina sequencing adaptor sequences (515F 5′-*ACACTCTTTCCCTACACGACGCTCTTCCGATCT*GTGYCAGCMGCCGCGGTAA-3′ and 806R 5′-*GTGACTGGAGTTCAGACGTGTGCTCTTCCGATCT*GGACTACNV GGGTWTCTAAT-3′; the italicized sequences are the Illumina sequences and the underlined sequences are the conserved bacterial primers 515F and 806R, respectively) followed by a second PCR reaction with the indexing primers as outlined in the Illumina 16S Metagenomic Sequencing Library Preparation guide [19] using the dual-index strategy [20]. All primers were purchased from Integrated DNA Technologies as 100 nmol standard desalted oligonucleotides. PCR reactions were performed in triplicates that consisted of Master Mix Platinum Hot Start (Thermo Fisher Scientific), 5 ng DNA template and 200 nM of each primer, with initial denaturation at 94 °C for 3 min followed by 35 cycles of denaturation at 94 °C for 45 s, annealing at 50 °C for 60 s, extension at 72 °C for 60 s and final extension at 72 °C for 10 min. After thermocycling, triplicate reactions were combined and diluted 50-fold. Indexes were then added in a PCR reaction that consisted of Master Mix Platinum Hot Start (Thermo Fisher), 400 nM of p5 indexing primer and 400 nM of p7 indexing primer, with initial denaturation at 94 °C for 1 min followed by 10 cycles of denaturation at 94 °C for 15 s, annealing at 64 °C for 15 s, extension at 72 °C for 60 s and final extension at 72 °C for 3 min. PCR products were purified using Agencourt AMPure beads (Beckman Coulter, Indianapolis, IN, USA) and were quantified using the Quant-iT PicoGreen dsDNA Assay Kit (Invitrogen – Molecular Probes, Eugene, OR, USA). Purified PCR products were diluted to a final concentration of 1 ng/µL, pooled in equal amounts and sequenced on an Illumina MiSeq instrument with the MiSeq Reagent Kit V2 (500 cycle; 2 × 250 bp paired-end reads) at the Center for Integrated Biosystems at Utah State University.

Demultiplexed paired-end reads were imported to the Quantitative Insights Into Microbial Ecology (QIIME2) version 2019.7 and quality-controlled with the DADA2 package [21] truncating forward and reverse reads to 240 and 230 base pairs, respectively, which removed sequences that contained Q scores below 30. The amplicon sequence variants were assigned taxonomy using the taxonomic classifier trained on the 515F/806R region of the Greengenes 13_8 database reference sequences [22] clustered at 99% sequence similarity. A multiple-sequence alignment was generated using MAFFT [23] and a phylogenetic tree was constructed using FastTree and midpoint-root. Core metric analyses were performed using the q2-diversity plugin with rarefication to a sampling depth of 29,000. Alpha-diversity measures included Faith’s phylogenetic diversity and Shannon’s diversity index. Beta-diversity measures included unweighted UniFrac distances. Principal coordinates analysis (PCoA) plot was generated using Emperor [24] from unweighted UniFrac distances [25]. 

#### 2.5.4. FMO3 Genotype

DNA was extracted from buffy coat collected at baseline using the DNeasy Tissue kit (Qiagen) according to the manufacturer’s protocol. The most common variant *FMO3* G472A (rs 2266782; E158K) known to influence N-oxygenation of TMA [26] was determined for each participant using a commercially available fluorescent Taqman endpoint SNP genotyping assay kit (Thermo Scientific) on the QuantStudio 12K Flex System (Applied Biosystems, Foster City, CA, USA). A reaction plate was created with 20 ng of dried-down genomic DNA on the 384-well optical reaction plate with initiation at 95 °C for 5 min followed by 45 cycles of denaturation at 95 °C for 15 s and annealing/extension at 60 °C for 60 s. A pre-read and post-PCR read were performed at 60 °C on the plate. Samples were run in duplicate reactions with 2 in-run standards and 6 negative water controls.

### 2.6. Statistical Analyses

Statistical analyses were conducted in SAS Version 9.3 (SAS Institute, Cary, NC, USA). Two-way repeated measures analysis of variance (ANOVA) using the PROC MIXED model procedure was performed to determine the effect of study meal, time and study meal-by-time interaction on metabolite concentration change from baseline. When a meal-and-time interaction was statistically significant, one-way ANOVA using the PROC MIXED model procedure was followed by the Tukey-Kramer post hoc test to determine the effect of study meal at each time of measurement. The incremental area under the curve (iAUC) was calculated for plasma TMAO response to study meal consumption across the 6-h study period. Participants were categorized as high-TMAO producers or low-TMAO producers based on the median excretion of TMAO (40%) in response to choline bitartrate consumption. Age, BMI, study session order and genotype *FMO3* G472A known to influence the conversion from trimethylamine to TMAO [27] were included in the initial statistical model. Variables that did not reach a significance of *p* ≤ 0.1 were removed to preserve degrees of freedom. 

Group significance for alpha-diversity and beta-diversity was assessed with Kruskal-Wallis and permutational multivariate analysis of variance (PERMANOVA) statistical framework using the Adonis function (999 permutations) in the vegan package of R [28], respectively. The analysis of composition of microbiomes (ANCOM) statistical framework [29] was used to identify taxa that are present in different abundances in high-versus low-TMAO producers at genus level. The W-value generated by ANCOM is a count of a number of sub-hypotheses that were detected to be significantly different across tested groups for a given taxon. Multiple hypothesis testing was corrected by the Benjamini-Hochberg False Discovery Rate (FDR) correction at 5%. Significant differences were reported at *p* < 0.05. All data are expressed as means ± SEM. 

## 3. Results

### 3.1. Participant Characteristics

Thirty-seven men participated in this study, of whom were 25 normal-weight men (with mean age of 25.3 ± 0.6 years, BMI of 23.3 ± 0.3 kg/m^2^) and 12 obese men (with mean age of 28.3 ± 2.0 years, BMI of 32.8 ± 0.7 kg/m^2^) as shown in Table 1. Due to the insufficient sample size of women (with only 4 normal-weight women) and obese individuals (12 obese men versus 25 normal-weight men), our final analyses pooled 37 men (25 normal-weight and 12 obese men together) to explore the role of gut microbiota composition in variations of TMAO response, thus our results are preliminary. Serum blood chemistry and blood cell counts were within the normal range. Fifty-one percent of the participants were homozygous wild-type GG genotype for *FMO3* G472A, 32% were heterozygous GA and 16% were homozygous variant AA (normal-weight: 48% GG, 36% GA and 16% AA; obese: 58% GG, 25% GA and 17% AA), whereby the percentage of participants with the variant genotypes resembled the approximate distribution observed in the general population [30].

### 3.2. Food Choline, Betaine and TMAO Content

The study meals consisted of 1 cup (237 mL) of tomato soup containing different forms of choline and provided approximately 600 mg of choline as choline bitartrate or phosphatidylcholine, compared to no choline control. Table 2 shows food choline, betaine and TMAO content, with total choline content calculated as the sum of free choline, glycerophosphocholine, phosphocholine, phosphatidylcholine, and sphingomyelin. The choline bitartrate meal had 619 mg total choline of which 590 mg was free choline and 14 mg phosphatidylcholine. The phosphatidylcholine meal had 623 mg total choline of which 19 mg was free choline and 588 mg phosphatidylcholine. A control meal with no choline added had 42 mg total choline of which 17 mg was free choline and 11 mg phosphatidylcholine. All study meals had the same betaine content of 7 mg. No TMAO was detected in all three meals.

### 3.3. TMAO Response to Choline Bitartrate and Phosphatidylcholine

**Figure 2:**
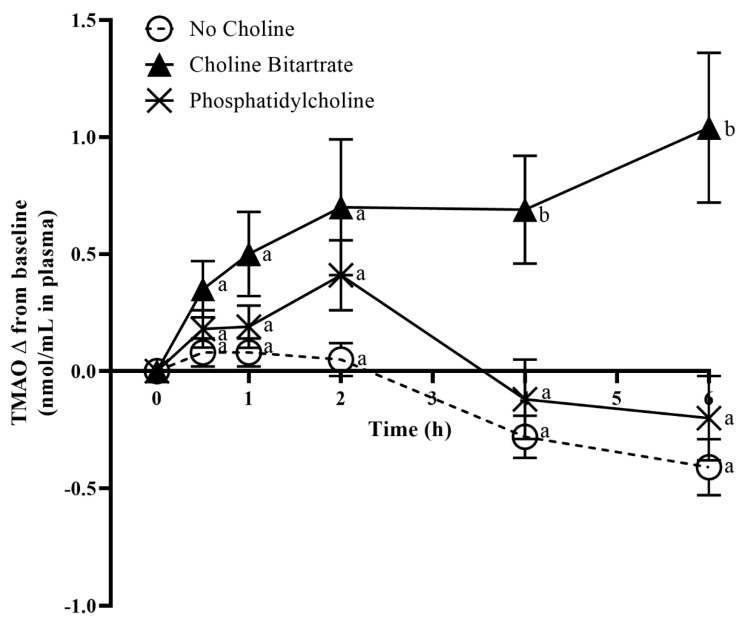
Effects of the study meals on plasma trimethylamine-*N*-oxide (TMAO) change from 0 min study-baseline across the 6-h study period. Different letter superscripts show a significant effect of study meal at each time point determined by one-way ANOVA, Tukey-Kramer post hoc test. Values are mean ± SEM, *n* = 37 per study meal.

**Figure 3:**
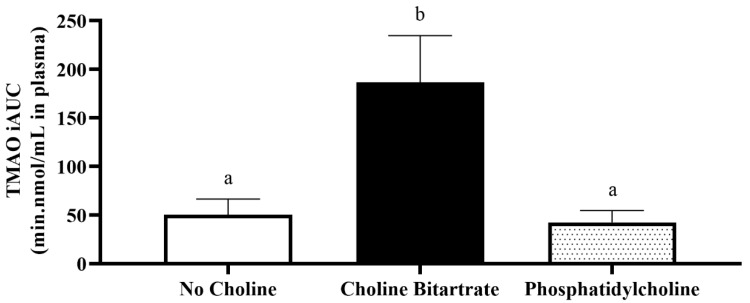
Plasma trimethylamine-*N*-oxide (TMAO) response to study meal consumption as incremental area under the curve (iAUC) across the 6-h study period. Different letter superscripts show a significant effect of study meal (one-way ANOVA, Tukey-Kramer post hoc test; *p* = 0.01). Values are mean ± SEM, *n* = 37 per study meal.

Fasting TMAO concentrations in plasma and urine at 0 min study-baseline were not different across the study meals. Study meal choline form significantly affected post-meal increases in plasma TMAO change from 0 min study-baseline (meal x time; *p* = 0.0002) as shown in Figure 2. Compared to phosphatidylcholine and no choline control, choline bitartrate yielded three-times higher plasma TMAO AUC (*p* = 0.01; Figure 3) and 4.4-times higher plasma TMAO maximum increase from baseline (*p* < 0.0001; Figure 2). 

In contrast, phosphatidylcholine did not differ in TMAO increase from baseline throughout the 6-h study period compared to no choline control. Similar to plasma TMAO change, choline bitartrate resulted in 2.5-times higher urinary TMAO change from 0 min study-baseline (*p* = 0.01) compared to phosphatidylcholine and no choline control, with no difference between phosphatidylcholine and no choline as shown in Table 3. 

**Figure 4:**
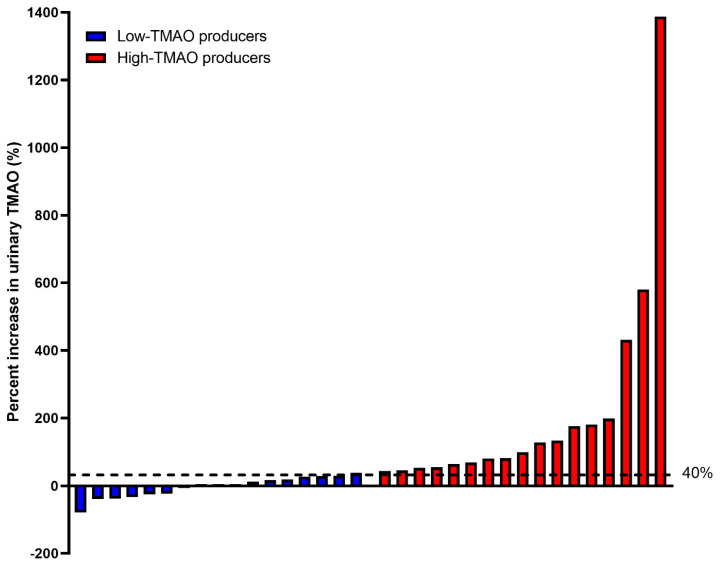
Percentage change in urinary trimethylamine-*N*-oxide (TMAO) concentrations following choline bitartrate consumption shown in rank-order of response. The percentage change ranged from −80% to 1400% among participants. The median value of 40% was used as a cutoff to stratify the participants into high-TMAO producers (*n* = 17; those with ≥40% urinary TMAO increase from 0 min study-baseline in response to choline bitartrate) and low-TMAO producers (*n* = 17; those with <40% urinary TMAO increase from 0 min study-baseline in response to choline bitartrate) were compared for the gut microbiome analyses.

The individual variations in urinary TMAO change from 0 min study-baseline after choline bitartrate consumption ranged from −80% to 1400% as shown in Figure 4. Forty percent increase was chosen as the median to stratify our participants into high-TMAO producers (*n* = 17; those with ≥40% urinary TMAO increase from 0 min study-baseline in response to choline bitartrate) versus low-TMAO producers (*n* = 17; those with <40% urinary TMAO increase from 0 min study-baseline in response to choline bitartrate). This value was chosen prior to the gut microbiome analyses, which retained 34 samples after rarefication to a sampling depth of 29,000 (see Section 3.5 on Gut Microbiota Composition).

### 3.4. Choline Response to Choline Bitartrate and Phosphatidylcholine

**Figure 5:**
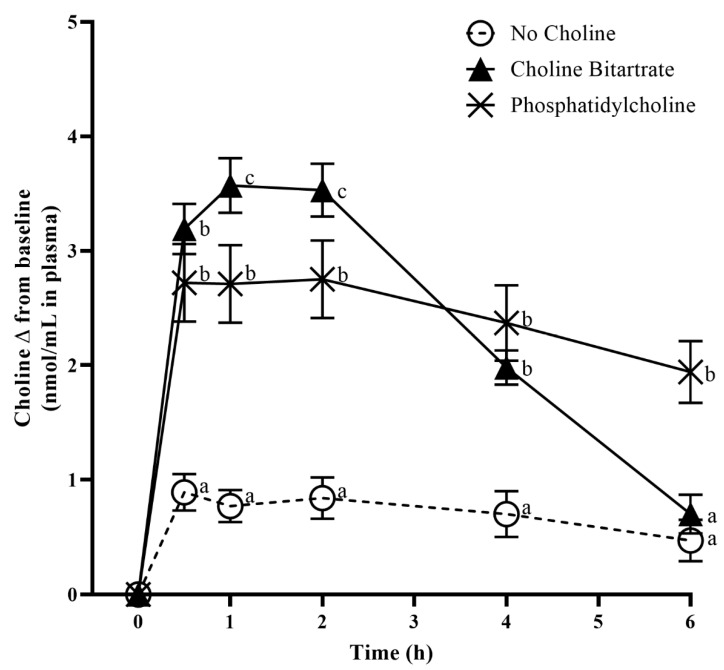
Effects of the study meals on plasma free choline change from 0 min study-baseline across the 6-h study period. Different letter superscripts show a significant effect of study meal at each time point determined by one-way ANOVA, Tukey-Kramer post hoc test. Values are mean ± SEM, *n* = 37 per study meal.

Fasting choline concentrations in plasma and urine at 0 min study-baseline were not different across the study meals. Choline form significantly influenced post-meal increases in plasma choline change from 0 min study-baseline (meal × time; *p* < 0.0001) as shown in Figure 5. Plasma free choline increase from 1–2 h was highest with choline bitartrate consumption (3.4-times greater) followed by phosphatidylcholine (2.4 times greater) compared to no choline control (*p* < 0.0001). At 6-h, plasma choline increase was 3.1-times greater only with phosphatidylcholine (*p* < 0.0001) with no differences between no choline control and choline bitartrate (Figure 5). Plasma free choline concentration peaked 1-h after choline bitartrate consumption and declined from 2–6 h reaching a level not different to that of no choline control at 6-h. The trajectory over time differed for phosphatidylcholine where plasma free choline concentration was not as high as that of choline bitartrate at the earlier time points but remained elevated throughout the study period, being greater than choline bitartrate and no choline groups at 6-h. Similar to plasma choline change, urinary choline change from study-baseline was 1.2-times higher after choline bitartrate and phosphatidylcholine consumption (*p* = 0.0005) compared to no choline control as shown in Table 3. 

### 3.5. Gut Microbiota Composition

Demultiplexing and DADA2 package within QIIME2 yielded a total of 7,590,141 high-quality gene sequences with mean sequence lengths of 292 ± 8 (mean ± standard deviation). Of the 37 samples, rarefication to a sampling depth of 29,000 retained 34 samples, which were used for downstream analyses. High-TMAO producers (*n* = 17; those with ≥40% urinary TMAO increase from 0 min study-baseline in response to choline bitartrate) and low-TMAO producers (*n* = 17; those with <40% urinary TMAO increase from 0 min study-baseline in response to choline bitartrate) were compared for the gut microbiome analyses.

**Figure 6:**
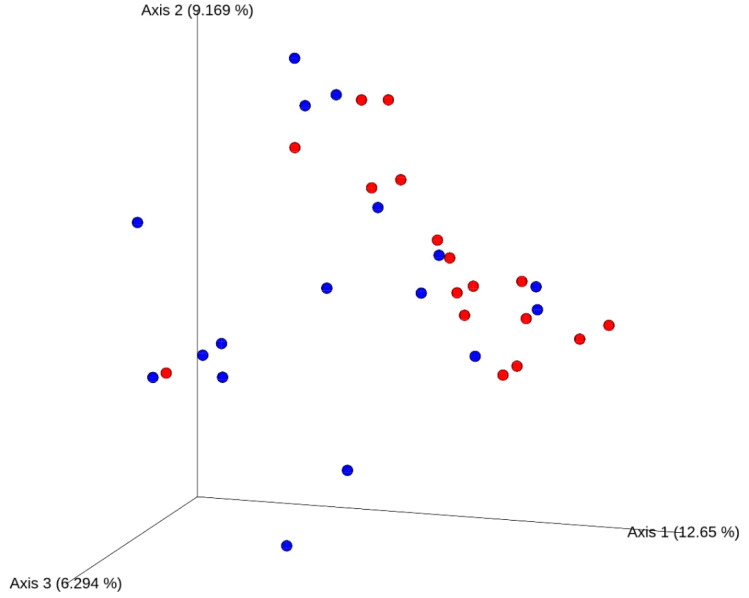
Principal coordinates analysis (PCoA) plot of the unweighted UniFrac distances from baseline stool samples of healthy male participants (*n* = 34). High-trimethylamine-*N*-oxide (TMAO) producers in red (*n* = 17; those with ≥40% urinary TMAO increase from 0 min study-baseline in response to choline bitartrate) and low-TMAO producers in blue (*n* = 17; those with <40% urinary TMAO increase from 0 min study-baseline in response to choline bitartrate) were significantly different from each other (Permutational multivariate analysis of variance, PERMANOVA *p* = 0.01, *R*^2^ = 0.05 with 999 permutations using the Adonis function).

Alpha-diversity (within-individual) measures were not different between high- versus low-TMAO producers (data not shown). High-TMAO producers had significantly different beta-diversity measures using the unweighted UniFrac distances (PERMANOVA *p* = 0.01, *R*^2^ = 0.05 with 999 permutations using the Adonis function) compared to low-TMAO producers as shown in Figure 6. 

Analysis of Composition of Microbiomes (ANCOM) revealed that high-TMAO producers had more abundant lineages of *Clostridium* from *Ruminococcaceae* (W = 11) and *Lachnospiraceae* (W = 8) in phylum *Firmicutes* compared to low-TMAO producers (*p* < 0.05 with the strength of the ANCOM test indicated by W-statistic) as shown in Table 4. ANCOM tests also revealed minor differences between high-TMAO and low-TMAO producers (W = 1–2), whereby high-TMAO producers were represented by *Oscillospira* and *Alistipes* whereas low-TMAO producers represented by *S24–7*, *Lactococcus*, *Christensenellaceae*, *Clostridiaceae*, *Bacteroidales*, *YS2, Catenibacterium, Gemella, Butyricicoccus* and *Ruminococcaceae*. The complete percentile abundances of taxa at genus level and W-statistics for high-TMAO versus low-TMAO producers are shown in Supplementary Table S1. 

## 4. Discussion

The findings support our hypothesis that consumption of choline bitartrate, but not phosphatidylcholine, leads to greater TMAO production and that gut microbiota composition contributes to alterations in TMAO response to choline bitartrate. The present study design incorporated measures of TMAO and choline metabolite absorption and excretion in conjunction with gut microbiota composition, and thus considered together dietary and physiological factors influencing TMAO metabolism. We demonstrate for the first time that heightened TMAO response to choline bitartrate intake may largely be attributed to abundant lineages of *Clostridium* (in phylum *Firmicutes*) and may provide guidance to personalized therapeutic strategies in reducing chronic disease risk. 

Choline bitartrate yielded 3-times higher plasma TMAO AUC and 4.4-times higher plasma TMAO maximum increase from baseline compared to phosphatidylcholine and no choline control, consistent with previous studies demonstrating that excess gut microbial-generated TMA production occurred with choline chloride [5,6]. Further, a recent study comparing choline bitartrate and krill oil containing phosphatidylcholine and omega-3 fatty acids also showed greater TMAO production only with choline bitartrate, but not krill oil [31]. The increase in TMAO concentration with choline bitartrate suggests that the non-ester form without requiring an enzymatic conversion step may be a preferred substrate for the gut microbial conversion to TMA and subsequent hepatic oxidation to TMAO. Choline bitartrate may be readily available and utilized as indicated by circulating free choline that peaked at 1-h higher, but the level was not sustained 2-h after consumption. The decline in plasma choline concentrations occurred concurrently with elevated plasma TMAO from 4–6 h, indicating the time required for the gut microbial conversion of TMA to TMAO. Higher urinary choline and TMAO excretion closely matched plasma response, and effective urinary TMAO clearance may indicate protection against the accumulation of TMAO. 

In contrast, TMAO change from study-baseline and AUC after phosphatidylcholine consumption did not differ compared to that of no choline control despite higher circulating and urinary free choline concentrations. The rate of free choline availability may be a primary determinant of TMAO production in that absorption of free choline, while rapid, is known to be saturated at 4 mM [32] leaving the excess to reach the large intestine, whereas a delayed conversion from phosphatidylcholine to free choline may limit large utilization of choline as a gut microbial substrate. Phosphatidylcholine consumption led to a sustained elevation of free choline concentration throughout the 6-h study period that appears to have entered the bloodstream at a slower rate than choline bitartrate. Given that the majority of choline in food is in the form of phosphatidylcholine, the absence of TMAO elevation with phosphatidylcholine counters arguments that dietary phosphatidylcholine should be avoided for TMAO-producing characteristics. Our findings are consistent with the previous long-term egg ingestion studies showing that the consumption of 3 eggs/day (~400 mg total choline/day) for four weeks resulted in higher plasma choline concentration without influencing plasma TMAO concentration and scavenger receptor gene expression compared to choline bitartrate supplementation [33]. Similarly, the consumption of two eggs/day for four weeks resulted in higher dietary and plasma choline concentrations, and improved markers of cardiovascular disease risk without affecting TMAO concentrations compared to an oatmeal breakfast [34]. However, greater TMAO concentration after a single-meal egg ingestion has also been reported [1,7]. The discrepancy may be due to phosphatidylcholine and food matrix interactions leading to different choline kinetics and individual differences contributing to TMAO response. 

An interindividual variation in urinary TMAO response to choline bitartrate consumption ranged widely from −80% to 1400% and this difference may be attributed to the gut microbiota composition. Consistent with our previous reports indicating that TMAO appeared to be a function of the gut microbiota differences between individuals [9], high-TMAO producers had significantly different gut microbiota composition compared to low-TMAO producers. Those individuals who had ≥40% increase in TMAO response after choline bitartrate consumption (high-TMAO producers) had more lineages of *Clostridium* from *Ruminococcaceae* and *Lachnospiraceae* compared to those who had <40% increase in TMAO response (low-TMAO producers), suggesting certain microbial populations may be responsible for a heightened TMAO response to dietary precursor intake. Both our present and previous studies [9] indicated that high-TMAO producers were represented by *Clostridiales* within the *Firmicutes* phylum, with a possibility that TMAO responses differentiated at genus level depending on dietary precursor intake and interaction with other nutrients. Moreover, our data support previous reports showing that microbial conversion of choline to TMA is catalyzed by choline TMA-lyase that is encoded by *Cut* gene clusters present in phyla *Firmicutes*, *Proteobacteria* and *Actinobacteria* [35]. Other minor differences in gut microbiota composition including *Oscillospira* and *Alistipes* abundant in high-TMAO producers and *S24-7*, *Lactococcus*, *Christensenellaceae*, *Clostridiaceae*, *Bacteroidales*, *YS2, Catenibacterium, Gemella, Butyricicoccus* and *Ruminococcaceae* abundant in low-TMAO producers may be related to differences in choline metabolism. Choline consumption by the intestinal microbiota may substantially contribute to choline deficiency [36], which in addition to genetics and other physiologic factors may contribute to individual differences in choline requirements. Although beta-diversity was significantly different, we did not observe differences in alpha-diversity measures, which may have been due to the presence of closely related features within our pool of participants. 

This study was a post hoc analysis of male participants, and provided some limitations in interpretation of the data. We explored whether differences in gut microbiota composition contributed to differences in TMAO production, which served as a rationale for our initial focus on obesity differences. Given a small sample size, our findings are limited to males in the normal-weight and obese BMI categories, thus may not be generalizable to underweight and overweight individuals, women, or those with pre-conditions of cardiovascular disease. TMAO response to choline bitartrate consumption or variability in response did not differ between normal-weight and obese individuals (data not shown) but a larger study may demonstrate TMAO accumulation in relation to underlying differences in metabolic responsiveness in obesity [37,38]. The *FMO3* G472A genotype was not an influential covariate, likely due to a small sample size of the individuals with the variant allele. Factors contributing to variability in TMAO response should be further examined, including development of a model that incorporates obesity, gut microbiome, genotype, background diet and physiological measures. In addition, the gut microbiome results should be interpreted with caution as differences in process, storage and exposure to ambient air during home collection of stool may have resulted in variability, even though participants were provided with standardized instruction and collection supplies. Another weakness of our study is the lack of clinical endpoints including assessment of cardiovascular disease risk. The mechanism by which TMAO may contribute to cardiovascular disease development involves enhanced cholesterol accumulation in macrophages and foam cell formation [2,39]. No changes in high-sensitivity C-reactive protein and oxidized low-density lipoprotein concentrations after a single meal-ingestion of eggs were previously reported despite higher TMAO concentrations [7]. Future studies using metabolomics and metagenomics analyses would delineate the functional relationships among choline, TMAO and atherosclerosis including host mechanisms in tissues (adipose tissue, muscle and liver). Lastly, this study determined an acute metabolic response and did not take into consideration adaptive changes in gut microbiota composition nor habitual consumption of dietary precursors and potential interaction with other nutrients. Long-term controlled feeding studies addressing the role of diet, gut microbiota and individual characteristics in TMAO production would fully assess the utility of TMAO as a prognostic marker for disease.

Our findings are significant on two fronts in: (1) clarifying the role of choline in TMAO metabolism as a key first step in determining physiological consequences of TMAO on long-term health and (2) identifying potential individual characteristics that increase propensity to TMAO accumulation that may guide the development of therapeutic strategies through precision nutrition. Choline is an essential bioactive micronutrient that has a critical role in biological processes including membrane biosynthesis, cholinergic neurotransmission and methylation reactions important for downstream gene expression and genome stability [40]. Prolonged choline deficiency is known to contribute to fatty liver disease and cognitive decline [41]. Moreover, emerging evidence highlights that maternal choline supplementation may improve placental function [42,43] and infant information processing speed [44] as well as reducing neonatal response to stress [42]. Recommendations proposing to restrict consumption of choline as a TMAO-generating nutrient require careful consideration and balanced reporting of dietary factors contributing to TMAO production. The development of individualized dietary recommendations based on gut microbiota composition may be a promising means to reduce risk of cardiovascular disease.

## Figures and Tables

**Table 1:** Participant characteristics for normal-weight (*n* = 25) and obese (*n* = 12) participants.

Participant Characteristics		Normal-Weight (*n* = 25)	Obese (*n* = 12)
Age	y	25.3 ± 0.6	28.3 ± 2.0
BMI	kg/m^2^	23.3 ± 0.3	32.8 ± 0.7
Genotype *FMO3* G472A rs 226678	GG%	48	58
	GA%	36	25
	AA%	16	17
Serum Blood Chemistry			
Glucose	mg/dL	87 ± 1	90 ± 2
BUN	mg/dL	16 ± 1	15 ± 1
Cr	mg/dL	1.0 ± 0.0	0.9 ± 0.0
Total bilirubin	mg/dL	0.9 ± 0.1	0.9 ± 0.3
Total protein	g/dL	7.1 ± 0.1	7.3 ± 0.3
ALP	IU/L	68 ± 3	74 ± 5
AST	IU/L	21 ± 1	23 ± 1
ALT	IU/L	21 ± 1	26 ± 2
eGFR	mL/min/1.73 m^2^	106 ± 3	109 ± 4
Blood Cell Counts			
RBC	×10^6^/µL	5.2 ± 0.1	5.4 ± 0.1
WBC	×10^3^/µL	5.9 ± 0.4	6.9 ± 0.4
Lymphocytes	×10^3^/µL	1.9 ± 0.1	2.3 ± 0.2
Monocytes	×10^3^/µL	0.5 ± 0.0	0.6 ± 0.0
Neutrophils	×10^3^/µL	3.2 ± 0.3	3.5 ± 0.3

Abbreviations: BMI, body mass index; *FMO3*, flavin-containing monooxygenase isoform 3; BUN, blood urea nitrogen; Cr, creatinine; ALP, alkaline phosphatase; AST, aspartate aminotransferase; ALT, alanine aminotransferase; eGFR, estimated glomerular filtration rate; RBC, red blood cell; and WBC, white blood cell. Values are mean ± SEM.

**Table 2:** Food amount and metabolite content of trimethylamine-*N*-oxide (TMAO), total choline, free choline, phosphatidylcholine and betaine in tomato soup containing (i) 600 mg choline as choline bitartrate; (ii) 600 mg choline as phosphatidylcholine; or (iii) no choline control.

		Tomato Soup with No Choline	Tomato Soup with 600 mg Choline as Choline Bitartrate	Tomato Soup with 600 mg Choline as Phosphatidylcholine
Food Amount	mL	237	237	237
Food metabolite content				
TMAO	mg	ND	ND	ND
Total choline ^1^	mg	42	619	623
Free choline	mg	17	590	19
Phosphatidylcholine	mg	11	14	588
Betaine	mg	7	7	7

^1^ Total choline amount was calculated as the sum of free choline, glycerophosphocholine, phosphocholine, phosphatidylcholine, and sphingomyelin. ND denotes not detectable. Values are shown as mean with each meal run in triplicates with the intraassay CV: 2% for free choline, 7% for phosphatidylcholine and 3% betaine.

**Table 3:** Effects of the study meals on urinary concentrations of trimethylamine-*N*-oxide (TMAO) and choline adjusted for creatinine (Cr) change from 0 min study-baseline. One-way ANOVA, Tukey-Kramer post hoc test showed a significant effect of study meal for TMAO and choline change from baseline as indicated by different letter superscripts. Values are mean ± SEM, *n* = 37 per study meal.

(nmol/mmol Cr in Urine)	No Choline	Choline Bitartrate	Phosphatidylcholine	*p* Value
TMAO	2.3 ± 1.4 ^a^	9.6 ± 2.2 ^b^	2.9 ± 1.7 ^a^	*p* = 0.01
Free choline	0.5 ± 0.1 ^a^	1.3 ± 0.2 ^b^	0.9 ± 0.1 ^b^	*p* = 0.0005

**Table 4:** Analysis of Composition of Microbiomes (ANCOM) results showing percentile abundances of taxa at genus level and W-statistics for high-trimethylamine-*N*-oxide (TMAO) producers (*n* = 17; those with ≥40% urinary TMAO increase from 0 min study-baseline in response to choline bitartrate) versus low-TMAO producers (*n* = 17; those with <40% urinary TMAO increase from 0 min study-baseline in response to choline bitartrate).

	High-TMAO Producers					Low-TMAO Producers						
Taxa	0%	25%	50%	75%	100%	0%	25%	50%	75%	100%	W	Reject Null Hypothesis
k__Bacteria; p__Firmicutes; c__Clostridia; o__Clostridiales; f__Ruminococcaceae; g__Clostridium	1	1	1	78	158	1	1	1	1	1	11	TRUE
k__Bacteria; p__Firmicutes; c__Clostridia; o__Clostridiales; f__Lachnospiraceae; g__Clostridium	87	152	263	385	1138	1	37	96	194	340	8	TRUE
k__Bacteria; p__Bacteroidetes; c__Bacteroidia; o__Bacteroidales; f__S24-7; g__	1	1	1	1	5	1	1	1	49	100	2	TRUE
k__Bacteria; p__Firmicutes; c__Bacilli; o__Lactobacillales; f__Streptococcaceae; g__Lactococcus	1	1	1	1	1	1	1	1	1	68	2	TRUE
k__Bacteria; p__Firmicutes; c__Clostridia; o__Clostridiales; f__Christensenellaceae; g__	1	1	1	1	125	1	1	8	90	420	2	TRUE
k__Bacteria; p__Firmicutes; c__Clostridia; o__Clostridiales; f__Clostridiaceae; g__	1	1	1	1	1	1	1	1	1	67	2	TRUE
k__Bacteria; p__Bacteroidetes; c__Bacteroidia; o__Bacteroidales; f__; g__	1	1	1	1	9	1	1	1	1	1876	2	TRUE
k__Bacteria; p__Cyanobacteria; c__4C0d-2; o__YS2; f__; g__	1	1	1	1	1	1	1	1	1	126	2	TRUE
k__Bacteria; p__Firmicutes; c__Erysipelotrichi; o__Erysipelotrichales; f__Erysipelotrichaceae; g__Catenibacterium	1	1	1	1	1	1	1	1	1	83	2	TRUE
k__Bacteria; p__Firmicutes; c__Clostridia; o__Clostridiales; f__Ruminococcaceae; g__Oscillospira	1	80	143	354	1359	53	124	162	264	432	1	TRUE
k__Bacteria; p__Firmicutes; c__Bacilli; o__Gemellales; f__Gemellaceae; g__Gemella	1	1	1	1	5	1	1	1	1	10	1	TRUE
k__Bacteria; p__Bacteroidetes; c__Bacteroidia; o__Bacteroidales; f__Rikenellaceae; g__Alistipes	1	90	167	485	6008	90	150	154	233	478	1	TRUE
k__Bacteria; p__Firmicutes; c__Clostridia; o__Clostridiales; f__Ruminococcaceae; g__Butyricicoccus	1	1	1	56	166	1	1	36	85	175	1	TRUE
k__Bacteria; p__Firmicutes; c__Clostridia; o__Clostridiales; f__Ruminococcaceae; g__	1	85	254	661	1205	1	196	340	653	2396	1	TRUE

Values are abundance of reads for each percentile within the high-TMAO producer versus low-TMAO producer groups. W-statistic indicates the strength of the ANCOM test (all at *p* < 0.05).

## Supplementary Materials

The following are available online at https://www.mdpi.com/2072-6643/12/8/2220/s1, Table S1: Percentile abundances of taxa at genus level and W-statistics for high-TMAO producers (*n* = 17; those with ≥40% urinary TMAO increase from 0 min study-baseline in response to choline bitartrate) versus low-TMAO producers (*n* = 17; those with <40% urinary TMAO increase from 0 min study-baseline in response to choline bitartrate).

## Abbreviations

Cr	creatinine
FMO3	Flavin-containing monooxygenase 3
iAUC	incremental area under the curve
LC-MS/MS	liquid chromatography-tandem mass spectrometry
PCoA	principal coordinate analysis
TMA	trimethylamine
TMAO	trimethylamine-N-oxide
